# Low prevalence of human enteropathogenic *Yersinia* spp. in brown rats (*Rattus norvegicus*) in Flanders

**DOI:** 10.1371/journal.pone.0175648

**Published:** 2017-04-12

**Authors:** Lieze Oscar Rouffaer, Kristof Baert, Anne-Marie Van den Abeele, Ivo Cox, Gerty Vanantwerpen, Lieven De Zutter, Diederik Strubbe, Katleen Vranckx, Luc Lens, Freddy Haesebrouck, Michel Delmée, Frank Pasmans, An Martel

**Affiliations:** 1 Department of Pathology, Bacteriology and Avian Diseases, Faculty of Veterinary Medicine, Ghent University, Merelbeke, Belgium; 2 Research Institute for Nature and Forest (INBO), Brussels, Belgium; 3 Microbiology Laboratory, AZ Sint Lucas Ghent, Ghent, Belgium; 4 Department of Veterinary Public Health and Food Safety, Faculty of Veterinary Medicine, Ghent University, Merelbeke, Belgium; 5 Department of Biology, Antwerp University, Antwerp, Belgium; 6 Applied Maths NV, Sint-Martens-Latem, Belgium; 7 Terrestrial Ecology Unit, Department of Biology, Ghent University, Ghent, Belgium; 8 Institute of Experimental and Clinical Research, Université Catholique de Louvain, Brussels, Belgium; Institut National de la Recherche Agronomique, FRANCE

## Abstract

Brown rats (*Rattus norvegicus*) have been identified as potential carriers of *Yersinia enterocolitica* and *Y*. *pseudotuberculosis*, the etiological agents of yersiniosis, the third most reported bacterial zoonosis in Europe. Enteropathogenic *Yersinia* spp. are most often isolated from rats during yersiniosis cases in animals and humans, and from rats inhabiting farms and slaughterhouses. Information is however lacking regarding the extent to which rats act as carriers of these *Yersinia* spp.. In 2013, 1088 brown rats across Flanders, Belgium, were tested for the presence of *Yersinia* species by isolation method. Identification was performed using MALDI-TOF MS, PCR on chromosomal- and plasmid-borne virulence genes, biotyping and serotyping. *Yersinia* spp. were isolated from 38.4% of the rats. Of these, 53.4% were designated *Y*. *enterocolitica*, 0.7% *Y*. *pseudotuberculosis* and 49.0% other *Yersinia* species. Two *Y*. *enterocolitica* possessing the *virF-*, *ail-* and *ystA-*gene were isolated. Additionally, the *ystB*-gene was identified in 94.1% of the other *Y*. *enterocolitica* isolates, suggestive for biotype 1A. Three of these latter isolates simultaneously possessed the *ail-*virulence gene. Significantly more *Y*. *enterocolitica* were isolated during winter and spring compared to summer. Based on our findings we can conclude that brown rats are frequent carriers for various *Yersinia* spp., including *Y*. *pseudotuberculosis* and (human pathogenic) *Y*. *enterocolitica* which are more often isolated during winter and spring.

## Introduction

Yersiniosis was the third most commonly reported bacterial zoonotic disease in Europe in 2013, causing illness in 1.92 out of 100 000 inhabitants [1]. The etiologic agents are human pathogenic *Yersinia enterocolitica* biotype (BT) 1B and 2–5, which possess chromosomally encoded virulence genes and carry the pYV (plasmid for *Yersinia* virulence), and to a minor extent *Y*. *pseudotuberculosis* [1;2]. *Y*. *enterocolitica* BT1A is most commonly regarded as non-pathogenic and often possesses the chromosomally encoded *ystB-*gene [3]. Wildlife has increasingly been recognized as reservoir and/or vector for various zoonotic diseases [4]. Especially rodents, such as the brown rat (*Rattus norvegicus*), have been appointed as potential carriers of pathogenic *Yersinia* spp.. Since brown rats are considered synanthropic rodents [5] they can be a possible source of infection for humans and other animals [6–9]. To evaluate food safety and human health risks, most of the studies on prevalence and epidemiology of *Yersinia* spp. in small mammals have therefore been conducted during yersiniosis outbreaks in animals and humans, in urban areas, in the surroundings of (pig) farms and in slaughterhouses [8–12]. Although epidemiologically important, information is lacking regarding the extent to which rats represent a potential reservoir of human pathogenic *Y*. *enterocolitica* and *Y*. *pseudotuberculosis*.

In this study we assessed the prevalence of *Y*. *enterocolitica* and *Y*. *pseudotuberculosis* in brown rats across Flanders, Belgium, not specifically related to disease outbreaks or to cities, with the aim to evaluate the contribution of brown rats as carriers of these *Yersinia* spp..

## Materials and methods

Within the framework of a rodenticide resistance study conducted by the Research Institute for Nature and Forest (INBO), in 2013, a total of 1088 brown rats were caught across Flanders, Belgium, by certified pest control operators of the Flanders Environment Agency (VMM) using wire mesh live traps measuring 50 length x 15 width x 13 height (cm). Most brown rats were captured on public land and occasionally on private land when oral permission was granted by the respective land owners. The brown rats were humanely killed by a percussive blow on the head (Directive 2010/63/EU; Annex IV) in the context of the pest control as stated by the Belgian legislation concerning animal protection and welfare (KB 14/08/86 art.15). According to the same legislation (KB 14/08/86 art.3.15) the killing of animals, only for the use of their organs and tissues, is not considered as an animal experiment. Therefore an approval of an ethical committee, as foreseen by the Belgian legislation concerning the protection of laboratory animals (KB 29/05/13), was not required. The brown rat is considered a major pest species which is legally controlled, for the trapping and killing of the rats no legal permits were required. Most individuals (71%) were captured in rural areas. The capture occasions were predominantly during the spring period (76.8%), while 18.1% of the rats were caught during summer, 2.7% during fall and 2.5% during winter. Capture dates were missing for 37 rats.

All the trapped individuals were kept frozen (-20°C) until April 2014, after which 0.5g of colon content was collected for the isolation of *Yersinia* spp.. Cold (4°C) enrichment was performed for three weeks using a 1/10 dilution of colon content in Phosphate Buffered Saline supplemented with 0.5% Peptone, 1% Mannitol and 0.15% Bile Salts (PMB). Before plating out onto cefsulodin–irgasan-novobiocin (CIN)-agar (Bio-rad, UK), an alkali treatment was performed using a 1/10 dilution of PMB-sample in KOH-solution (0.25%KOH, 0.75%NaCl) which was vortexed for 20 seconds. The CIN plates were incubated for 24 hours at 30°C, and reassessed after being kept at room temperature for 24 hours. Suspicious colonies were purified onto MacConkey agar (Oxoid, Hampshire, UK). MALDI-TOF MS (Matrix-Assisted Laser Desorption Ionization- Time-of-Flight Mass Spectrometry), was performed using the Bruker Daltonik MALDI Biotyper, at the Department of Clinical Microbiology, Laboratory Medicine, AZ Sint-Lucas in Ghent. The samples were cultured for 24 hours at 30°C on Columbia agar with sheep blood (Oxoid, Wesel, Germany). One colony per sample was smeared upon a MALDI steel target plate, covered with 1 μl α-cyano-4-hydroxycinnamic acid (HCCA) matrix and, after air drying, loaded into the MALDI-Biotyper. Mass Spectrometry detections were carried out with Maldi biotyper 3.0 RTC software in standard IVD settings, using the 5627 reference strains library. Every MALDI-TOF assigned-*Y*. *enterocolitica* and *Y*. *pseudotuberculosis* was tested for the presence of chromosomal- (*ail*, *inv*, *ystA*, *ystB*) and pYV-plasmid-borne (*virF*) virulence genes [2]. Positive controls, *Y*. *enterocolitica* BT1A (FAVV208), human pathogenic *Y*. *enterocolitica* 4/O:3 (75.55b), and *Y*. *pseudotuberculosis* (22.36a), were provided by the Department of Veterinary Public Health and Food Safety of Ghent University. When *Y*. *enterocolitica* harboured the *ystB-*gene in combination with the *ail-*gene, the latter was sequenced and analyzed using Basic Local Alignment Search Tool (BLAST) [13]. *Ail-*positive *Y*. *enterocolitica* isolates were bioserotyped and *Y*. *pseudotuberculosis* isolates were serotyped at the National Reference Center *Yersinia* (IREC) and at the Department of Veterinary Public Health and Food Safety of Ghent University. The MALDI-TOF profiles were compared against the biotypes with linear discriminant analysis (LDA) to try to identify discriminating peaks [14].

To test whether *Y*. *enterocolitica* prevalence differed between seasons, we applied a Generalized Linear Model (GLM) with *Y*. *enterocolitica* presence or absence as binary dependent variable and season as independent predictor variable, using a binomial error distribution. To account for possible spatial autocorrelation in *Y*. *enterocolitica* prevalence, latitude and longitude of capture locations were forced into the model as fixed effects, fixed predictor variables [15]. To test for differences in prevalence between seasons, contrasts were set up using the general linear hypothesis test (glht)- function of the R library ‘multcomp’ (multiple comparisons), resulting in Bonferroni-corrected p-values adjusted for multiple testing [16]. All analyses were conducted in R [17].

## Results and discussion

**Fig 1:**
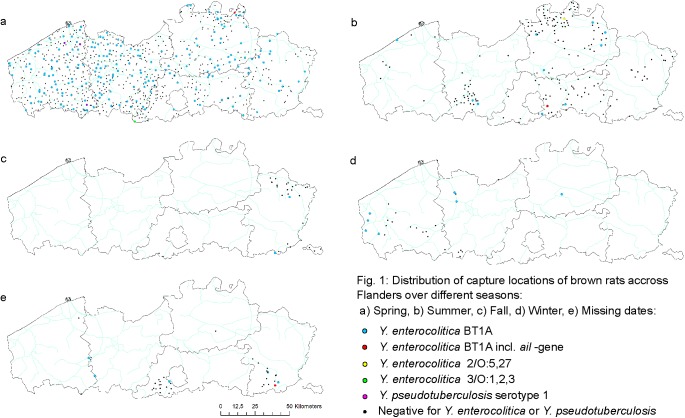
Distribution of capture locations of brown rats across Flanders over different seasons.

In Flanders, *Yersinia* spp. were isolated from 418 out of 1088 (38.4%) brown rats tested (Fig 1), which, in 13 individuals, harbored more than one *Yersinia* spp.. Of these *Yersinia* spp., 53.4% (223/418) were designated *Y*. *enterocolitica*, 0.7% (3/418) *Y*. *pseudotuberculosis*, and 49.0% (205/418) (Table 1) other *Yersinia* species.

**Table 1:** Total number of rats testing positive for *Yersinia*, in the respective seasons.

	Spring	Summer	Fall	Winter	Total number of *Yersinia* isolates
*Y*. *enterocolitica*	196^a^	12^b^	2	7	217*
*Y*. *pseudotuberculosis*	3	0	0	0	3
*Yersinia* species	163	25	6	5	199*
Total number of brown rats examined	807	190	28	26	

^a^ Including *Y*. *enterocolitica* BT 3/O:1,2,3 and one *ail* positive *Y*. *enterocolitica* BT 1A

^b^ Including *Y*. *enterocolitica* BT 2/O:5,27 and one *ail* positive *Y*. *enterocolitica* BT 1A

* Dates of 37 brown rats were missing, six of which were identified as *Y*. *enterocolitica* (incl. one *ail* positive *Y*. *enterocolitica* BT 1A) and six environmental *Yersinia* spp..

MALDI-TOF MS and PCR on the combination of chromosomal- and plasmid-borne virulence genes have previously been used for the identification of (enteropathogenic) *Y*. *enterocolitica* and *Y*. *pseudotuberculosis* [2;18–23]. Although MALDI-TOF MS has proven to accurately perform species specific identification of *Yersinia* spp. [19;21], the sensitivity and specificity of the technique highly depends on the validation of the reference library used to identify the different species [19;24]. Since only *Y*. *enterocolitica* and *Y*. *pseudotuberculosis* were accurately validated in the Bruker Daltonik MALDI Biotyper at the Department of Clinical Microbiology, resulting in high specificity and sensitivity [21;24], only these species will be further discussed. The results of the MALDI-TOF MS-identification of the *Yersinia* species other than *Y*. *enterocolitica* and *Y*. *pseudotuberculosis* have been provided within the supporting information (S1 Table), although these results have to be interpreted with caution due to the lack of validation of the MALDI-TOF reference database for the other *Yersinia* spp. [19;24].

Due to the psychrotolerant nature of *Yersinia* spp., the observed prevalence could be expected to vary among seasons [25]. Indeed, our study found a significantly higher prevalence of *Y*. *enterocolitica* in brown rats during winter (26.9%) and spring (24.3%) months, compared to summer (6.3%) (P-values = 0.007 and <0.001 respectively) (Table 2). This observation is in line with previous studies in rodents and other animals [6;11;26;27], although a high prevalence of *Y*. *enterocolitica* during the summer has also been reported in rats [10].

**Table 2:** Seasonal comparisons of *Y*. *enterocolitica* prevalence.

	Estimate	Std. Error	z value	Pr(>|z|)
spring—fall = = 0	1.4306	0.7571	1.890	0.20901
summer—fall = = 0	-0.1705	0.8005	-0.213	0.99611
winter—fall = = 0	1.6211	0.8883	1.825	0.23658
summer—spring = = 0	-1.6011	0.3164	-5.060	< 0.001
winter—spring = = 0	0.1905	0.4561	0.418	0.97220
winter—summer = = 0	1.7916	0.5519	3.246	0.00535

Tukey’s post-hoc tests for multiple contrasts were used to establish Bonferroni-corrected significant differences in *Y*. *enterocolitica* prevalence between seasons. Results show that compared to the summer period, *Y*. *enterocolitica* prevalence was higher in the spring and in the winter. Prevalence did not significantly differ between other season comparisons.

The prevalence of *Y*. *enterocolitica* (20.5% = 223/1088) in brown rats is similar to other studies in rodents [28]. In the vast majority (93.3% = 208/223), the presence of the *ystB-*gene was demonstrated, which has been inferred to be restricted to BT1A [2;18;29]. Of these *ystB* positive isolates, three possessed an additional *ail-*virulence gene (100% identity with Accession number: FR847859.1). Controversy exists about the pathogenicity of *Y*. *enterocolitica* BT1A. Since most BT1A strains do not possess the typical virulence plasmid pYV, lack the chromosomal virulence genes such as the *ail-*gene and are often isolated from the environment [3;22], BT1A has been regarded as non-pathogenic. However, the increasing isolation of this biotype from clinical cases draws more attention to BT1A [3;22]. Although rare, the presence of the *ail-*gene has previously been demonstrated in BT1A isolates [20;27]. The presence of the enterotoxin-gene *ystB* in combination with the *ail-*virulence gene could be an indication that these BT1A strains possess virulent characteristics [30]. However, potential loss of gene function, related to horizontal gene transfer cannot be ruled out [20]. The high genotypic diversity of BT1A makes the classification in clinical and non-clinical isolates more problematic since other, yet unknown, virulence factors could be contributing to the observed virulence in some strains [3;22;31].

Although the majority of *Y*. *enterocolitica* belonged to the supposedly non-pathogenic BT1A, two human pathogenic *Y*. *enterocolitica* (bioserotype 2/O:5,27 and 3/O:1,2,3) [1;32], possessing the *virF-*, *ail-* and *ystA-*gene virulence gene, were isolated from rats living in the proximity of livestock farms. These results are in line with other studies, indicating that *Y*. *enterocolitica* BT1A is widespread in rodents, but human pathogenic bioserotypes are rather rare [27;28;33]. The recovery of human pathogenic *Y*. *enterocolitica* from rodents could be related to the presence of farmhouses, as was hypothesized for bioserotype 4/O:3 and 3/O:3, for which the presence in rodents is presumed to be related to pig farms and pig slaughterhouses [9;10;23;27]. Despite the isolation of the two human pathogenic bioserotypes in the proximity of livestock farms, no definitive conclusion can be made from this observation, since the animals on the respective farms were not tested for the presence of pathogenic *Y*. *enterocolitica*. Furthermore, bioserotype 2/O:5,27 has been isolated from a variety of animals, such as cattle, pigs, hares and wild boars, and no primary reservoir has been identified yet [34;35]. Bioserotype 3/O:1,2,3, alternatively called the “chinchilla-type” [36], has also previously been isolated from pigs [37]. No peaks discriminating between the different biotypes could be identified in the MALDI-TOF spectra (data not shown).

Three (0.3%) *Y*. *pseudotuberculosis* serotype I possessing the *inv-* and *virF*-virulence gene, the most frequently isolated *Y*. *pseudotuberculosis* in Europe, were isolated. This serotype has been reported to cause disease in humans and other animals, such as birds and rats [12;38–40]. The low percentage of *Y*. *pseudotuberculosis* observed in our study is in line with the absence or sporadic detection of *Y*. *pseudotuberculosis* in rodents in other studies [8;9;27;28].

Although a large number of brown rats was screened for the presence of enteropathogenic *Yersinia* spp. in Flanders, the additional investigation of other wild living animals, as potential carriers or reservoirs for enteropathogenic *Yersinia*, could substantially improve our knowledge on the epidemiology and ecology of these pathogens and the potential risk these animals pose on farm-animals and human health.

In conclusion, our results demonstrate that rats are frequent carriers for *Yersinia* spp. such as non-pathogenic and human pathogenic *Y*. *enterocolitica* and *Y*. *pseudotuberculosis*.

## Supporting information

S1 Table MALDI-TOF results of *Yersinia* spp. other than *Y*. *enterocolitica* and *Y*. *pseudotuberculosis*. (DOCX)
