# Supplementary material for: Low prevalence of human enteropathogenic Yersinia spp. in brown rats (Rattus norvegicus) in Flanders
Source: PLoS One. 2017 Apr 12;12(4):e0175648. doi: 10.1371/journal.pone.0175648 (PMC5389835; doi:10.1371/journal.pone.0175648)
Supplement: S1 Table — (DOCX) [file pone.0175648.s001.docx]

| **S1 Table.** **MALDI-TOF results of *Yersinia* spp. other than *Y. enterocolitica* and *Y. pseudotuberculosis*** | | | | | | | | |
| --- | --- | --- | --- | --- | --- | --- | --- | --- |
| N° | x | y | Date | MALDI-TOF  Result 1 | Score 1 | MALDI-TOF  Result 2 | Score 2 | Consistency/Mismatch |
| 1 | 163600 | 229400 | 20/08/2013 | *Y. frederiksenii* | 2.087 | *Y. enterocolitica* | 1.949 | B/SM |
| 2 | 181263 | 192602 | 4/04/2013 | *Y. enterocolitica* | 2.215 | *Y. kristensenii* | 2.176 | B/SM |
| 3 | 171950 | 235100 | 31/07/2013 | *Y. enterocolitica* | 2.146 | *Y. intermedia* | 2.110 | B/SM |
| 4 | 217425 | 172725 |  | *Y. intermedia* | 2.219 | *Y. enterocolitica* | 2.187 | B/SM |
| 5 | 234575 | 176825 | 27/05/2013 | *Y. enterocolitica* | 2.158 | *Y. kristensenii* | 2.127 | B/SM |
| 6 | 228500 | 179840 | 6/06/2013 | *Y. frederiksenii* | 2.203 | *Y. enterocolitica* | 2.163 | B/SM |
| 7 | 160200 | 237000 | 23/07/2013 | *Y. enterocolitica* | 2.161 | *Y. frederiksenii* | 2.117 | B/SM |
| 8 | 199253 | 230750 | 15/05/2013 | *Y. intermedia* | 2.388 | *Y. enterocolitica* | 2.166 | B/SM |
| 9 | 161700 | 231600 | 26/08/2013 | *Y. intermedia* | 2.224 | *Y. enterocolitica* | 2.198 | B/SM |
| 10 | 188000 | 202000 | 10/04/2013 | *Y. intermedia* | 2.527 | *Y. enterocolitica* | 2.142 | B/SM |
| 11 | 189820 | 195180 | 22/05/2013 | *Y. enterocolitica* | 2.031 | *Y. kristensenii* | 1.934 | B/SM |
| 12 | 185000 | 229150 | 24/05/2013 | *Y. rhodei* | 2.345 | *Y. rhodei* | 2.262 | A/M |
| 13 | 150800 | 191100 | 22/04/2013 | *Y. kristensenii* | 2.474 | *Y. enterocolitica* | 2.368 | B/SM |
| 14 | 152100 | 235200 | 3/07/2013 | *Y. intermedia* | 2.497 | *Y. enterocolitica* | 2.210 | B/SM |
| 15 | 167300 | 228200 | 7/06/2013 | *Y. enterocolitica* | 2.007 | *Y. kristensenii* | 1.918 | B/SM |
| 16 | 154000 | 220700 | 29/07/2013 | *Y. frederiksenii* | 2.411 | *Y. frederiksenii* | 2.192 | B/SM |
| 17 | 184400 | 212900 | 5/07/2013 | *Y. intermedia* | 2.412 | *Y. enterocolitica* | 2.198 | B/SM |
| 18 | 177000 | 238000 | 14/08/2013 | *Y. enterocolitica* | 2.012 | *Y. frederiksenii* | 1.923 | B/SM |
| 19 | 159900 | 238100 | 27/06/2013 | *Y. intermedia* | 2.240 | *Y. enterocolitica* | 1.971 | B/SM |
| 20 | 151600 | 196000 | 29/04/2013 | *Y. enterocolitica* | 2.038 | *Y. enterocolitica* | 1.935 | A/M |
| 21 | 148200 | 221700 | 7/08/2013 | *Y. enterocolitica* | 1.935 | *Y. kristensenii* | 1.930 | B/SM |
| 22 | 176800 | 234000 | 5/07/2013 | *Y. intermedia* | 2.542 | *Y. enterocolitica* | 2.134 | B/SM |
| 23 | 199500 | 227500 | 8/07/2013 | *Y. rhodei* | 2.430 | *Y. rhodei* | 2.325 | A/M |
| 24 | 164400 | 230700 | 21/08/2013 | *Y. frederiksenii* | 2.273 | *Y. enterocolitica* | 2.202 | B/SM |
| 25 | 193600 | 215900 | 30/07/2013 | *Y. enterocolitica* | 2.167 | *Y. frederiksenii* | 2.126 | B/SM |
| 26 | 118150 | 163100 | 28/06/2013 | *Y. enterocolitica* | 2.229 | *Y. frederiksenii* | 2.192 | B/SM |
| 27 | 97080 | 159060 | 19/03/2013 | *Y. intermedia* | 2.202 | *Y. enterocolitica* | 2.149 | B/SM |
| 28 | 93760 | 166300 | 10/07/2013 | *Y. enterocolitica* | 2.146 | *Y. enterocolitica* | 1.942 | A/M |
| 29 | 110900 | 203600 | 25/07/2013 | *Y. intermedia* | 2.367 | *Y. enterocolitica* | 2.135 | B/SM |
| 30 | 165740 | 175300 | 1/07/2013 | *Y. enterocolitica* | 2.027 | *Y. aleksiciae* | 1.902 | B/SM |
| 31 | 135300 | 176600 | 27/06/2013 | *Y. intermedia* | 2.351 | *Y. enterocolitica* | 2.214 | B/SM |
| 32 | 218150 | 186350 | 15/05/2013 | *Y. frederiksenii* | 2.359 | *Y. frederiksenii* | 2.209 | B/SM |
| 33 | 128100 | 155600 | 9/07/2013 | *Y. frederiksenii* | 2.093 | *Y. enterocolitica* | 2.001 | B/SM |
| 34 | 167130 | 181190 | 7/06/2013 | *Y. intermedia* | 2.536 | *Y. intermedia* | 2.241 | B/SM |
| 35 | 155610 | 179120 | 18/06/2013 | *Y. enterocolitica* | 2.081 | *Y. enterocolitica* | 2.054 | B/SM |
| 36 | 97300 | 169720 | 1/08/2013 | *Y. enterocolitica* | 1.992 | *Y. kristensenii* | 1.821 | B/SM |
| 37 | 124250 | 159350 | 28/06/2013 | *Y. intermedia* | 2.313 | *Y. intermedia* | 2.076 | B/SM |
| 38 | 160660 | 179000 | 26/06/2013 | *Y. intermedia* | 2.199 | *Y. enterocolitica* | 2.146 | B/SM |
| 39 | 101420 | 164840 | 21/06/2013 | *Y. intermedia* | 2.396 | *Y. enterocolitica* | 2.213 | B/SM |
| 40 | 127800 | 154450 | 9/07/2013 | *Y. frederiksenii* | 2.227 | *Y. frederiksenii* | 2.055 | B/SM |
| 41 | 121525 | 156400 | 27/06/2013 | *Y. enterocolitica* | 1.969 | *Y. intermedia* | 1.886 | A/M |
| 42 | 95000 | 210700 | 9/04/2013 | *Y. mollaretii* | 2.207 | *Y. enterocolitica* | 2.167 | B/SM |
| 43 | 142160 | 161660 |  | *Y. enterocolitica* | 2.033 | *Y. frederiksenii* | 1.905 | B/SM |
| 44 | 62500 | 216900 | 23/04/2013 | *Y. intermedia* | 2.315 | *Y. intermedia* | 2.066 | B/SM |
| 45 | 65400 | 203500 | 8/05/2013 | *Y. intermedia* | 2.106 | *Y. intermedia* | 2.093 | B/SM |
| 46a | 99700 | 218500 | 26/03/2013 | *Y. kristensenii* | 2.471 | *Y. enterocolitica* | 2.416 | B/SM |
| 46b | 99700 | 218500 | 26/03/2013 | *Y. intermedia* | 2.163 | *Y. enterocolitica* | 2.054 | B/SM |
| 47a | 81000 | 213000 | 26/04/2013 | *Y. kristensenii* | 2.213 | *Y. enterocolitica* | 2.204 | B/SM |
| 47b | 81000 | 213000 | 26/04/2013 | *Y. enterocolitica* | 2.180 | *Y. frederiksenii* | 2.163 | B/SM |
| 48 | 136500 | 167260 |  | *Y. intermedia* | 2.445 | *Y. intermedia* | 2.241 | B/SM |
| 49 | 100900 | 203500 | 27/03/2013 | *Y. kristensenii* | 2.386 | *Y. enterocolitica* | 2.147 | B/SM |
| 50 | 97400 | 214900 | 16/05/2013 | *Y. mollaretii* | 2.114 | *Y. enterocolitica* | 2.079 | B/SM |
| 51 | 99500 | 198900 | 5/04/2013 | *Y. enterocolitica* | 2.051 | *Y. kristensenii* | 2.049 | B/SM |
| 52 | 73100 | 221600 | 24/04/2013 | *Y. kristensenii* | 2.179 | *Y. enterocolitica* | 2.149 | B/SM |
| 53 | 131400 | 159600 |  | *Y. mollaretii* | 2.132 | *Y. enterocolitica* | 2.091 | B/SM |
| 54 | 83200 | 209700 | 6/05/2013 | *Y. intermedia* | 2.216 | *Y. enterocolitica* | 2.083 | B/SM |
| 55 | 238800 | 165300 | 30/08/2013 | *Y. frederiksenii* | 2.057 | *Y. enterocolitica* | 2.020 | B/SM |
| 56 | 74700 | 220280 | 18/04/2013 | *Y. frederiksenii* | 2.526 | *Y. frederiksenii* | 2.285 | B/SM |
| 57 | 98200 | 195200 | 19/04/2013 | *Y. intermedia* | 2.356 | *Y. enterocolitica* | 2.156 | B/SM |
| 58 | 89200 | 203900 | 4/06/2013 | *Y. intermedia* | 2.414 | *Y. enterocolitica* | 2.164 | B/SM |
| 59 | 96200 | 207400 | 8/04/2013 | *Y. intermedia* | 2.409 | *Y. enterocolitica* | 2.182 | B/SM |
| 60 | 126123 | 173259 | 14/06/2013 | *Y. enterocolitica* | 2.088 | *Y. kristensenii* | 1.898 | B/SM |
| 61 | 72100 | 180000 | 4/06/2013 | *Y. intermedia* | 2.198 | *Y. intermedia* | 1.868 | A/M |
| 62 | 60300 | 170640 | 25/06/2013 | *Y. intermedia* | 2.449 | *Y. enterocolitica* | 2.140 | B/SM |
| 63 | 132122 | 166980 | 20/06/2013 | *Y. intermedia* | 2.226 | *Y. frederiksenii* | 1.825 | A/M |
| 64 | 112400 | 206900 | 26/06/2013 | *Y. enterocolitica* | 2.222 | *Y. frederiksenii* | 2.217 | B/SM |
| 65 | 78840 | 180060 | 21/06/2013 | *Y. intermedia* | 2.322 | *Y. enterocolitica* | 2.080 | B/SM |
| 66 | 129304 | 170965 | 14/06/2013 | *Y. intermedia* | 2.237 | *Y. enterocolitica* | 2.094 | B/SM |
| 67 | 137948 | 196073 | 28/05/2013 | *Y. intermedia* | 2.419 | *Y. enterocolitica* | 2.213 | B/SM |
| 68 | 129910 | 163115 | 20/06/2013 | *Y. enterocolitica* | 2.231 | *Y. frederiksenii* | 2.220 | B/SM |
| 69 | 194000 | 182000 | 9/08/2013 | *Y. enterocolitica* | 2.279 | *Y. frederiksenii* | 2.260 | B/SM |
| 70 | 173550 | 237250 | 29/04/2013 | *Y. enterocolitica* | 2.222 | *Y. kristensenii* | 2.145 | B/SM |
| 71 | 209560 | 160800 | 26/06/2013 | *Y. intermedia* | 2.080 | *Y. kristensenii* | 2.073 | B/SM |
| 72 | 191500 | 172080 | 13/06/2013 | *Y. kristensenii* | 2.024 | *Y. enterocolitica* | 1.979 | B/SM |
| 73 | 214369 | 197746 | 30/04/2013 | *Y. intermedia* | 2.286 | *Y. enterocolitica* | 2.213 | B/SM |
| 74 | 175150 | 235000 | 25/04/2013 | *Y. mollaretii* | 2.197 | *Y. kristensenii* | 2.019 | B/SM |
| 75 | 131065 | 188981 | 18/06/2013 | *Y. intermedia* | 2.463 | *Y. enterocolitica* | 2.140 | B/SM |
| 76 | 140586 | 197390 | 23/04/2013 | *Y. kristensenii* | 2.397 | *Y. enterocolitica* | 2.308 | B/SM |
| 77 | 64465 | 168920 | 25/06/2013 | *Y. intermedia* | 2.356 | *Y. enterocolitica* | 2.093 | B/SM |
| 78 | 78720 | 156660 | 25/06/2013 | *Y. intermedia* | 2.128 | *Y. enterocolitica* | 2.101 | B/SM |
| 79 | 147814 | 188073 | 28/05/2013 | *Y. enterocolitica* | 2.105 | *Y. bercovieri* | 1.984 | B/SM |
| 80 | 157300 | 237700 | 20/08/2013 | *Y. intermedia* | 2.432 | *Y. enterocolitica* | 2.181 | B/SM |
| 81a | 196000 | 235650 | 30/04/2013 | *Y. mollaretii* | 2.187 | *Y. kristensenii* | 1.987 | B/SM |
| 81b | 196000 | 235650 | 30/04/2013 | *Y. intermedia* | 2.186 | *Y. intermedia* | 1.962 | B/SM |
| 82 | 131600 | 205600 | 19/06/2013 | *Y. intermedia* | 2.319 | *Y. enterocolitica* | 2.216 | B/SM |
| 83 | 128300 | 212000 | 2/07/2013 | *Y. kristensenii* | 2.285 | *Y. enterocolitica* | 2.269 | B/SM |
| 84 | 133070 | 187311 | 21/06/2013 | *Y. intermedia* | 2.160 | *Y. intermedia* | 2.106 | B/SM |
| 85 | 61200 | 180500 | 5/03/2013 | *Y. enterocolitica* | 2.123 | *Y. enterocolitica* | 1.971 | A/M |
| 86 | 177000 | 229180 | 4/06/2013 | *Y. intermedia* | 2.203 | *Y. enterocolitica* | 2.042 | B/SM |
| 87 | 154000 | 237250 | 24/07/2013 | *Y. intermedia* | 2.313 | *Y. enterocolitica* | 2.142 | B/SM |
| 88 | 73800 | 211000 | 14/05/2013 | *Y. mollaretii* | 2.236 | *Y. enterocolitica* | 2.117 | B/SM |
| 89 | 198120 | 232650 | 2/05/2013 | *Y. intermedia* | 2.180 | *Y. enterocolitica* | 2.010 | B/SM |
| 90 | 61700 | 202600 | 17/05/2013 | *Y. intermedia* | 2.297 | *Y. enterocolitica* | 2.217 | B/SM |
| 91 | 195900 | 238135 | 22/04/2013 | *Y. rhodei* | 2.454 | *Y. rhodei* | 2.351 | A/M |
| 92 | 87900 | 197500 | 8/05/2013 | *Y. frederiksenii* | 2.183 | *Y. enterocolitica* | 2.147 | B/SM |
| 93 | 206770 | 187185 | 26/04/2013 | *Y. enterocolitica* | 2.354 | *Y. kristensenii* | 2.343 | B/SM |
| 94 | 182000 | 175250 | 13/08/2013 | *Y. intermedia* | 2.251 | *Y. enterocolitica* | 1.967 | B/SM |
| 95 | 177750 | 224650 | 12/06/2013 | *Y. intermedia* | 2.248 | *Y. enterocolitica* | 2.044 | B/SM |
| 96 | 232800 | 202000 | 28/10/2013 | *Y. intermedia* | 2.288 | *Y. enterocolitica* | 2.025 | B/SM |
| 97 | 230800 | 195700 | 22/07/2013 | *Y. kristensenii* | 2.306 | *Y. enterocolitica* | 2.242 | B/SM |
| 98 | 193460 | 178680 | 11/06/2013 | *Y. frederiksenii* | 2.594 | *Y. frederiksenii* | 2.401 | BSM |
| 99 | 200630 | 161340 | 21/08/2013 | *Y. enterocolitica* | 2.017 | *Y. frederiksenii* | 1.886 | B/SM |
| 100 | 70100 | 200000 | 5/06/2013 | *Y. intermedia* | 2.314 | *Y. enterocolitica* | 1.978 | B/SM |
| 101 | 193270 | 168000 | 24/05/2013 | *Y. kristensenii* | 2.445 | *Y. enterocolitica* | 2.344 | B/SM |
| 102 | 125184 | 170205 | 13/06/2013 | *Y. intermedia* | 2.327 | *Y. intermedia* | 1.999 | B/SM |
| 103 | 75800 | 174800 | 13/06/2013 | *Y. enterocolitica* | 2.076 | *Y. frederiksenii* | 2.076 | B/SM |
| 104 | 75100 | 201800 | 7/05/2013 | *Y. rhodei* | 2.304 | *Y. rhodei* | 2.273 | A/M |
| 105 | 109899 | 161090 | 9/04/2013 | *Y. enterocolitica* | 2.323 | *Y. kristensenii* | 2.269 | B/SM |
| 106 | 53900 | 196500 | 25/04/2013 | *Y. intermedia* | 2.275 | *Y. enterocolitica* | 2.244 | B/SM |
| 107 | 54200 | 187700 | 28/03/2013 | *Y. intermedia* | 2.272 | *Y. enterocolitica* | 2.003 | B/SM |
| 108 | 112489 | 168576 | 2/04/2013 | *Y. frederiksenii* | 2.202 | *Y. enterocolitica* | 2.185 | B/SM |
| 109 | 56000 | 194800 | 23/04/2013 | *Y. intermedia* | 2.230 | *Y. intermedia* | 1.998 | B/SM |
| 110 | 26100 | 180000 | 8/04/2013 | *Y. kristensenii* | 2.343 | *Y. kristensenii* | 2.264 | B/SM |
| 111 | 43600 | 181300 | 18/02/2013 | *Y. aleksiciae* | 2.099 | *Y. intermedia* | 1.993 | B/SM |
| 112 | 43600 | 202000 | 17/05/2013 | *Y. frederiksenii* | 1.935 | *Y. rhodei* | 1.712 | A/M |
| 113 | 116768 | 167346 | 29/03/2013 | *Y. mollaretii* | 2.184 | *Y. kristensenii* | 1.998 | B/SM |
| 114 | 50600 | 174800 | 8/04/2013 | *Y. intermedia* | 2.231 | *Y. enterocolitica* | 2.154 | B/SM |
| 115 | 34300 | 186400 | 27/03/2013 | *Y. enterocolitica* | 2.051 | *Y. kristensenii* | 2.019 | B/SM |
| 116 | 116719 | 170098 | 12/04/2013 | *Y. enterocolitica* | 1.838 | *Y. kristensenii* | 1.831 | B/M |
| 117 | 111587 | 168492 | 8/04/2013 | *Y. intermedia* | 1.974 | *Y. enterocolitica* | 1.952 | B/SM |
| 118 | 26600 | 188300 | 25/04/2013 | *Y. intermedia* | 2.310 | *Y. frederiksenii* | 1.951 | B/SM |
| 119 | 206125 | 185375 | 4/07/2013 | *Y. intermedia* | 2.250 | *Y. intermedia* | 2.127 | B/SM |
| 120 | 34480 | 184550 | 11/04/2013 | *Y. mollaretii* | 2.199 | *Y. enterocolitica* | 2.138 | B/SM |
| 121 | 24600 | 197300 | 2/04/2013 | *Y. intermedia* | 2.362 | *Y. intermedia* | 2.185 | B/SM |
| 122 | 116333 | 165453 | 28/03/2013 | *Y. intermedia* | 2.005 | *Y. intermedia* | 1.912 | B/SM |
| 123 | 47900 | 184100 | 10/02/2013 | *Y. enterocolitica* | 1.946 | *Y. enterocolitica* | 1.739 | A/M |
| 124 | 114260 | 172395 | 12/04/2013 | *Y. intermedia* | 2.181 | *Y. enterocolitica* | 2.049 | B/SM |
| 125 | 112207 | 161427 | 28/03/2013 | *Y. intermedia* | 2.220 | *Y. enterocolitica* | 2.052 | B/SM |
| 126 | 51000 | 178720 | 8/04/2013 | *Y. intermedia* | 2.073 | *Y. intermedia* | 2.019 | B/SM |
| 127 | 238600 | 208700 | 29/10/2013 | *Y. enterocolitica* | 1.967 | *Y. enterocolitica* | 1.866 | A/M |
| 128 | 173400 | 178880 | 26/08/2013 | *Y. enterocolitica* | 1.988 | *Y. enterocolitica* | 1.866 | A/M |
| 129 | 157220 | 172580 | 26/07/2013 | *Y. intermedia* | 2.088 | *Y. enterocolitica* | 2.003 | B/SM |
| 130 | 158900 | 175230 | 8/08/2013 | *Y. intermedia* | 2.362 | *Y. enterocolitica* | 2.213 | B/SM |
| 131 | 234500 | 162500 | 4/11/2013 | *Y. enterocolitica* | 2.153 | *Y. enterocolitica* | 2.018 | B/SM |
| 132 | 206350 | 182720 |  | *Y. intermedia* | 2.174 | *Y. enterocolitica* | 2.062 | B/SM |
| 133 | 235500 | 205000 | 28/10/2013 | *Y. enterocolitica* | 2.139 | *Y. enterocolitica* | 1.951 | A/M |
| 134 | 158900 | 196200 | 11/04/2013 | *Y. intermedia* | 2.155 | *Y. enterocolitica* | 2.148 | B/SM |
| 135 | 231700 | 209500 | 28/10/2013 | *Y. intermedia* | 2.040 | *Y. intermedia* | 2.034 | A/M |
| 136 | 177350 | 168300 | 20/08/2013 | *Y. intermedia* | 2.221 | *Y. enterocolitica* | 2.035 | B/SM |
| 137 | 243900 | 180300 | 24/10/2013 | *Y. intermedia* | 2.245 | *Y. enterocolitica* | 2.147 | B/SM |
| 138 | 97560 | 163440 | 20/03/2013 | *Y. intermedia* | 2.356 | *Y. intermedia* | 2.181 | B/SM |
| 139 | 113060 | 203000 | 30/04/2013 | *Y. intermedia* | 2.022 | *Y. enterocolitica* | 1.992 | B/SM |
| 140 | 141700 | 172850 | 26/06/2013 | *Y. mollaretii* | 2.148 | *Y. enterocolitica* | 1.949 | B/SM |
| 141 | 104500 | 166640 | 28/06/2013 | *Y. intermedia* | 2.175 | *Y. intermedia* | 2.114 | B/SM |
| 142 | 97100 | 160900 | 13/06/2013 | *Y. intermedia* | 2.247 | *Y. intermedia* | 2.235 | B/SM |
| 143 | 137300 | 217150 | 31/05/2013 | *Y. intermedia* | 1.977 | *Y. enterocolitica* | 1.934 | B/SM |
| 144 | 116800 | 202700 | 7/05/2013 | *Y. intermedia* | 2.116 | *Y. enterocolitica* | 1.933 | B/SM |
| 145 | 122370 | 210150 | 22/05/2013 | *Y. intermedia* | 2.283 | *Y. enterocolitica* | 2.126 | B/SM |
| 146a | 127100 | 198300 | 29/05/2013 | *Y. mollaretii* | 2.144 | *Y. aleksiciae* | 2.073 | B/SM |
| 146b | 127100 | 198300 | 29/05/2013 | *Y. enterocolitica* | 2.144 | *Y. frederiksenii* | 2.106 | B/SM |
| 147 | 124100 | 204500 | 16/04/2013 | *Y. intermedia* | 2.255 | *Y. enterocolitica* | 2.092 | B/SM |
| 148 | 101980 | 167620 | 3/07/2013 | *Y. intermedia* | 2.345 | *Y. enterocolitica* | 2.071 | B/SM |
| 149 | 173050 | 168400 | 8/08/2013 | *Y. intermedia* | 2.125 | *Y. enterocolitica* | 2.085 | B/SM |
| 150 | 111100 | 196130 | 24/04/2013 | *Y. enterocolitica* | 1.808 | *Y. enterocolitica* | 1.698 | B/M |
| 151 | 134167 | 169576 | 17/06/2013 | *Y. enterocolitica* | 2.046 | *Y. kristensenii* | 2.007 | B/SM |
| 152 | 117000 | 192200 | 18/04/2013 | *Y. intermedia* | 2.324 | *Y. enterocolitica* | 2.181 | B/SM |
| 153 | 102290 | 165680 | 21/06/2013 | *Y. intermedia* | 2.464 | *Y. enterocolitica* | 2.254 | B/SM |
| 154 | 140180 | 168800 |  | *Y. enterocolitica* | 1.987 | *Y. intermedia* | 1.951 | B/SM |
| 155 | 135700 | 217200 | 18/06/2013 | *Y. intermedia* | 2.236 | *Y. enterocolitica* | 2.192 | B/SM |
| 156 | 125800 | 194200 | 25/04/2013 | *Y. intermedia* | 2.223 | *Y. enterocolitica* | 2.114 | B/SM |
| 157 | 56380 | 163780 | 11/06/2013 | *Y. enterocolitica* | 2.153 | *Y. frederiksenii* | 2.141 | B/SM |
| 158 | 66550 | 162200 | 3/07/2013 | *Y. intermedia* | 2.129 | *Y. enterocolitica* | 2.004 | B/SM |
| 159 | 60250 | 177500 | 31/05/2013 | *Y. mollaretii* | 2.264 | *Y. enterocolitica* | 2.064 | B/SM |
| 160 | 68000 | 177800 | 21/06/2013 | *Y. intermedia* | 2.136 | *Y. intermedia* | 2.011 | B/SM |
| 161 | 97000 | 179100 | 30/05/2013 | *Y. enterocolitica* | 2.208 | *Y. kristensenii* | 2.164 | B/SM |
| 162 | 45400 | 195600 | 22/08/2013 | *Y. intermedia* | 2.280 | *Y. enterocolitica* | 2.174 | B/SM |
| 163 | 102000 | 184600 | 29/05/2013 | *Y. rhodei* | 2.450 | *Y. rhodei* | 2.181 | B/SM |
| 164 | 79950 | 187200 | 22/05/2013 | *Y. enterocolitica* | 2.133 | *Y. mollaretii* | 2.099 | B/SM |
| 165 | 36000 | 166500 | 28/06/2013 | *Y. intermedia* | 2.379 | *Y. enterocolitica* | 2.163 | B/SM |
| 166 | 50800 | 202600 | 28/06/2013 | *Y. enterocolitica* | 2.080 | *Y. intermedia* | 2.073 | B/SM |
| 167 | 98900 | 188500 | 24/05/2013 | *Y. bercovieri* | 2.315 | *Y. enterocolitica* | 2.130 | B/SM |
| 168 | 39485 | 206862 | 9/07/2013 | *Y. intermedia* | 2.451 | *Y. intermedia* | 2.173 | B/SM |
| 169 | 38080 | 169550 | 3/07/2013 | *Y. intermedia* | 2.488 | *Y. intermedia* | 2.271 | B/SM |
| 170 | 79600 | 190100 | 27/06/2013 | *Y. intermedia* | 2.374 | *Y. intermedia* | 2.264 | B/SM |
| 171 | 90500 | 180400 | 10/04/2013 | *Y. enterocolitica* | 2.142 | *Y. bercovieri* | 2.078 | B/SM |
| 172 | 68600 | 185800 | 13/06/2013 | *Y. intermedia* | 2.233 | *Y. intermedia* | 2.088 | B/SM |
| 173 | 95900 | 177750 | 30/05/2013 | *Y. intermedia* | 2.223 | *Y. intermedia* | 2.202 | B/SM |
| 174 | 58500 | 216140 | 20/06/2013 | *Y. intermedia* | 2.387 | *Y. enterocolitica* | 2.221 | B/SM |
| 175 | 59000 | 167000 | 5/07/2013 | *Y. intermedia* | 2.245 | *Y. enterocolitica* | 2.094 | B/SM |
| 176 | 55800 | 210500 | 24/06/2013 | *Y. mollaretii* | 2.357 | *Y. enterocolitica* | 2.093 | B/SM |
| 177 | 80700 | 194600 | 25/06/2013 | *Y. intermedia* | 2.146 | *Y. enterocolitica* | 2.141 | B/SM |
| 178 | 101500 | 186150 | 29/05/2013 | *Y. intermedia* | 2.201 | *Y. intermedia* | 2.169 | B/SM |
| 179 | 90340 | 169500 | 31/05/2013 | *Y. intermedia* | 2.289 | *Y. enterocolitica* | 2.219 | B/SM |
| 180 | 83280 | 171650 | 31/05/2013 | *Y. intermedia* | 2.258 | *Y. enterocolitica* | 2.151 | B/SM |
| 181 | 33350 | 171280 | 22/04/2013 | *Y. intermedia* | 2.214 | *Y. intermedia* | 2.121 | B/SM |
| 182 | 66400 | 187600 | 6/06/2013 | *Y. intermedia* | 2.196 | *Y. enterocolitica* | 2.187 | B/SM |
| 183 | 54500 | 172780 | 4/06/2013 | *Y. intermedia* | 2.386 | *Y. enterocolitica* | 2.195 | B/SM |
| 184 | 79600 | 191100 | 1/07/2013 | *Y. intermedia* | 2.354 | *Y. enterocolitica* | 2.158 | B/SM |
| 185 | 81150 | 158850 | 25/06/2013 | *Y. intermedia* | 2.295 | *Y. enterocolitica* | 2.239 | B/SM |
| 186 | 46020 | 162200 | 11/04/2013 | *Y. intermedia* | 2.310 | *Y. enterocolitica* | 2.209 | B/SM |
| 187 | 34420 | 175340 | 7/06/2013 | *Y. frederiksenii* | 2.453 | *Y. frederiksenii* | 2.427 | B/SM |
| 188 | 88000 | 175300 | 24/05/2013 | *Y. kristensenii* | 2.305 | *Y. enterocolitica* | 2.293 | B/SM |
| 189 | 36275 | 171650 | 24/05/2013 | *Y. enterocolitica* | 2.196 | *Y. intermedia* | 2.167 | B/SM |
| 190 | 95100 | 174700 | 31/05/2013 | *Y. frederiksenii* | 2.159 | *Y. enterocolitica* | 2.037 | B/SM |
| 191 | 99100 | 181600 | 30/05/2013 | *Y. rhodei* | 2.426 | *Y. rhodei* | 2.329 | B/SM |
| 192 | 81850 | 178800 | 22/05/2013 | *Y. rhodei* | 2.257 | *Y. rhodei* | 2.158 | A/M |
| 193 | 61000 | 168800 | 23/05/2013 | *Y. mollaretii* | 2.171 | *Y. enterocolitica* | 2.079 | B/SM |
| 194 | 61600 | 193500 | 21/06/2013 | *Y. intermedia* | 2.490 | *Y. enterocolitica* | 2.248 | B/SM |
| 195 | 82800 | 169300 | 10/04/2013 | *Y. intermedia* | 2.344 | *Y. enterocolitica* | 2.212 | B/SM |
| 196 | 41040 | 160000 | 23/04/2013 | *Y. intermedia* | 2.512 | *Y. intermedia* | 2.287 | B/SM |
| 197 | 58100 | 169750 | 5/06/2013 | *Y. intermedia* | 2.194 | *Y. intermedia* | 2.127 | B/SM |
| 198 | 74400 | 185600 | 21/06/2013 | *Y. intermedia* | 2.346 | *Y. enterocolitica* | 2.174 | B/SM |
| 199 | 78400 | 217400 | 25/07/2013 | *Y. intermedia* | 2.240 | *Y. enterocolitica* | 2.008 | B/SM |
| 200 | 34650 | 180590 | 17/05/2013 | *Y. aleksiciae* | 2.230 | *Y. kristensenii* | 1.964 | B/SM |
| 201 | 82700 | 191700 | 26/06/2013 | *Y. enterocolitica* | 2.201 | *Y. kristensenii* | 2.134 | B/SM |
| 202 | 104100 | 202700 | 21/06/2013 | *Y. intermedia* | 2.229 | *Y. enterocolitica* | 2.039 | B/SM |
| 203 | 66800 | 175200 | 14/06/2013 | *Y. frederiksenii* | 2.110 | *Y. enterocolitica* | 2.054 | B/SM |
| 204 | 105700 | 212000 | 15/06/2013 | *Y. rhodei* | 2.189 | *Y. rhodei* | 2.132 | B/SM |
| 205 | 83120 | 161520 | 5/06/2013 | *Y. intermedia* | 2.098 | *Y. intermedia* | 1.985 | A/M |

The top match and second best match (score 1 and 2 respectively), based on the mass spectra of a single spot for each isolate, are provided in the table. Results were assigned a consistency category based on the manufacturer’s criteria.

- Species consistency (A) was attributed to a result when all matches scoring ≥1.9 show to be of the same species, and all matches scoring ≥1.7 are of the same genus.
- Genus consistency (B) is characterized by a top match score between 1.899 and 1.7, or by matches scoring ≥1.9 but that are not of the same species, taking into account that all matches scoring ≥1.7 have to be of the same genus.
- No consistency (C) in identification occurs when the top match score is <1.7, or matches scoring ≥1.7 are not of the same genus.

For each individual identification, the top 10 of best matches was also carefully assessed for mismatches:

- Mismatches (M) were defined as results on the list of the 10 best matches that differ with the top match result at genus level or species level.
- Significant mismatches (SM) indicate results within the top 10 best matches that differ with the top match result at genus level (score ≥ 1.7) or species level (score ≥ 1.9).
